# A Novel Scoring System for AUS Thyroid Nodule

**DOI:** 10.1155/ije/6736469

**Published:** 2025-08-12

**Authors:** Nuha A. Alsaleh, Mohammed A. Alswayyed, Shatha A. Alduraywish, Budur T. Althobaiti, Beshayer A. Alhentti, Afnan A. Alzayed, Monirah M. Alsalouli, Abdullah I. Aljunaydil, Malak A. Alzahrani

**Affiliations:** ^1^Department of Surgery, College of Medicine, King Saud University, Riyadh, Saudi Arabia; ^2^Department of Pathology and Laboratory Medicine, College of Medicine, King Saud University, Riyadh, Saudi Arabia; ^3^Department of Community Medicine, College of Medicine, King Saud University, Riyadh, Saudi Arabia; ^4^Diagnostic Radiology Department, King Abdullah Specialized Children's Hospital, Riyadh, Saudi Arabia

**Keywords:** atypia of undetermined significance, nuclear atypia, nuclear score, risk of malignancy, subcategorization, the Bethesda system

## Abstract

**Objective:** The primary objective of this study was to further subclassify Bethesda III atypia of undetermined significance (AUS) cytological findings in thyroid lesions using fine-needle aspiration cytology (FNAC). The secondary objective was to propose a novel scoring system to estimate the malignancy risk within these subcategories.

**Methods:** We conducted a retrospective analysis of patients diagnosed with Bethesda III AUS who underwent thyroidectomy at King Khalid University Hospital, Riyadh, Saudi Arabia, from January 2017 to December 2024. Clinical, radiological, and pathological data—including FNAC slides and surgical specimens—were thoroughly reviewed. Cases classified as Bethesda III were further subclassified into six subtypes: III-A (small follicular pattern, poorly cohesive cells, and minimal colloid), III-B (nuclear and/or cellular atypia), III-C (cytological and architectural atypia), III-D (predominantly Hurthle cells), III-E (indeterminate atypia), and III-F (atypical lymphoid cells suggestive of possible lymphoma). A structured malignancy risk scoring system was developed by integrating cytological subtypes, radiologic features (microcalcifications, irregular margins, and hypervascularity), clinical risk factors (family history and prior radiation), and patient age. Statistical analysis was performed using SPSS Version 22, with chi-squared and Fisher's exact tests used to assess associations between variables and malignancy.

**Results:** A total of 338 cases were analyzed, with a mean age of 42 ± 5 years and a female predominance. The malignancy distribution by subcategory was as follows: III-A: 10.65% (*n* = 36), III-B: 54.73% (*n* = 185), III-C: 22.49% (*n* = 76), III-D: 5.03% (*n* = 17), III-E: 7.69% (*n* = 26), and III-F: 0.3% (*n* = 1). Significant associations were found between malignancy and both cytological subcategory and presence of high-risk ultrasound features (*p* < 0.05). The proposed scoring system stratified patients into three risk groups: low (scores 0–2), moderate (2.5–4), and high (≥ 4.5), offering a predictive framework for clinical decision-making.

**Conclusion:** This study highlights the heterogeneity within Bethesda III AUS nodules and supports subclassification as a meaningful step toward more accurate malignancy risk assessment. The proposed scoring system may serve as a practical tool to guide individualized management decisions. Further prospective validation is warranted.

## 1. Introduction

Thyroid nodules are common with a prevalence of 4%–7% [1]. Thyroid cancer in Saudi Arabia is ranked as the 2nd among women and the 9th among men [2, 3]. Ultrasound-guided fine-needle aspiration (FNA) is one of the most essential tools for investigating thyroid nodules [4]. FNA cytology has demonstrated high utility in diagnosing thyroid nodules. The Bethesda System for Reporting Thyroid Cytopathology (TBSRTC) is a universally utilized framework for interpreting thyroid FNAs [5]. Interobserver variability is significant for indeterminate categories due to subjective morphological interpretation. One of the most perplexing categories is Bethesda category III. TBSRTC 3rd edition is organized and subdivided into subcategories as atypia of undetermined significance (AUS) “nuclear” and AUS “other” in the recent edition of TBSRTC [6, 7].

However, the distinct interpretations and individual approaches within this category, coupled with the absence of established standards, contribute to its extensive utilization. This phenomenon has resulted in a diversity of opinions among cytopathologists [8]. In addition, it is difficult to determine the ROM since only a tiny portion of AUS cases has surgical follow-ups. The predicted mean ROM for the AUS category reported in the TBSRTC 3rd edition is 22 (13%–30%) [8–10].

Many authors have emphasized subcategorizing the AUS category due to its wide range of malignancy risks and heterogeneous nature. They have reported higher malignancy rates in AUS with nuclear or cytological atypia than in AUS with architectural atypia [11]. Finally, the recent 3rd edition of TBSRTC recommends a two-tiered subcategorization of AUS, including AUS “nuclear” and AUS “other” [9].

Numerous researchers have underscored the importance of subcategorizing the AUS category, given its diverse malignancy risks and heterogeneous characteristics. Studies have indicated that AUS cases with nuclear or cytological atypia exhibit higher malignancy rates compared with those with architectural atypia [11]. Clinical and radiological findings, along with the Bethesda classification, are crucial for informed clinical decision-making. Therefore, we propose to develop a new subclassification and a scoring system. Limited published data exist on integrating clinical, radiological, and AUS factors into a coherent scoring system.

## 2. Methods

The study included a detailed review of clinical information and radiological findings from patients' electronic medical records, pathological samples (surgical specimens and FNA slides), and postoperative pathological reports for all cases. Comparisons between malignancy rates and in different categories were performed using chi-squared and Fisher's exact tests. Statistical analysis will be performed using Statistical Package for the Social Sciences (SPSS) Version 22 software (SPSS Inc., Chicago, IL, USA).

### 2.1. Study Subject Selection

#### 2.1.1. Inclusion Criteria

• All patients with FNA Bethesda III who underwent thyroidectomy at King Khalid University Hospital (KKUH).
• Patients whose preoperative examinations, surgery, and postoperative evaluations were all performed at KKUH.
• The availability of FNA material for review.

#### 2.1.2. Exclusion Criteria

• Patients who lack the reported patient data standardization.
• There is an absence of a definitive match between the FNA area and the postoperative tissue samples.
• Those with a known diagnosis of thyroid carcinoma (previously submitted to partial thyroidectomy).
• All other Bethesda classes.

### 2.2. Study Procedures

A detailed review of clinical and radiological findings from patients' electronic medical records, pathological samples (surgical specimens and FNA slides), and pathological reports for all cases was performed.

### 2.3. Data Collection Method/Data Source

The study includes a detailed review of clinical and radiological findings from patients' electronic medical records, pathological samples (surgical specimens and FNA slides), and pathological reports for all cases.

The research team reviewed patients' files in accordance with the established standards of the local Institutional Review Board (Ethics Committee). All thyroid FNA Bethesda III reports produced during the same period were reviewed. Data were collected for each patient based on multiple follow-up FNAs during the study period. A thyroid pathologist reviewed a random sample of 20 cases with Bethesda III FNA specimens for confirmation. The FNA diagnosis was blinded to the pathologist to avoid bias. Then, all patients who underwent surgical resection (thyroidectomy) for the FNA-confirmed Bethesda III nodule were included. Final FNA with Bethesda III reports from each patient were correlated to the postoperative findings as benign versus malignant.

Patients diagnosed as Bethesda III are cytologically subdivided into the following six subcategories:

• Bethesda III-A is characterized by a small follicular pattern with poorly attached cells and a small amount of colloid.
• Bethesda III-B is characterized by the presence of cellular and nuclear atypia, regardless of the distribution pattern of the cells.
• Bethesda III-C: cytologic and architectural atypia.
• Bethesda III-D: Hurthle cell aspirates.
• Bethesda III-E: atypia not otherwise specified.
• Bethesda III-F: atypical lymphoid cells to rule out lymphoma.

The preoperative ultrasounds for all patients were reviewed for the following radiological findings:

• Microcalcification
• Hypervascularity
• Irregularity

The patient's medical records were reviewed for family history and prior radiation therapy.

• The postoperative pathology was reviewed to determine whether it was benign or malignant. The data were analyzed, and a scoring system was developed to integrate all the above data and predict malignancies.

## 3. Result

The Institutional Review Board of King Saud University was reviewed. Participants' anonymity was ensured by assigning each participant a code number for analysis only, and confidentiality was granted.

Demographic and clinical characteristics of the study participants (*N* = 338), showing age distribution, gender ratio, family history of thyroid cancer, and prior radiation exposure. The data highlight a predominance of female patients (84.32%) with a mean age of 43.3 ± 12.3 years. Approximately 11.54% had a family history of thyroid cancer, and 2.37% had a history of radiation therapy (Table 1).

Comparison of clinical and radiological characteristics was performed according to the tumor type (benign vs. malignant) in patients with Bethesda III AUS thyroid nodules (*N* = 338).  Table 2 outlines the distribution of gender, age, family history, prior radiation, and ultrasound features (microcalcification, hypervascularity, and irregularity) across benign (59.47%) and malignant (40.53%) cases. While none of the variables reached statistical significance (*p* > 0.05), trends suggest potential associations warranting further investigation, particularly the presence of microcalcifications and a first-degree family history (Table 2).

### 3.1. Statistical Analysis

Comparisons between malignancy rates in different categories were performed using chi-squared and Fisher's exact tests. Statistical analysis was performed using SPSS Version 22 software (SPSS Inc., Chicago).

Four hundred cases were collected, with a mean age of 42 ± 5 y, with a female predominance of 285 (84.32%) females vs. 53 (15.68%) males. Common radiological findings included hypervascularity (50.30%) and microcalcifications (28.70%), essential indicators in the thyroid nodule assessment (Tables 3 and 4).

Comparisons between malignancy rates in different categories were performed using chi-squared and Fisher's exact tests. The rates of differentiated thyroid cancer were 5.6% in Category A, 33.7% in Category B, 38.7% in Category C, 11.2% in Category D, 7.8% in Category E, and 3% in Category F.

The difference in malignancy rates was insignificant among categories A, E, and F. The chi-square test for tumor type yields a *p* value of 0.086. This result is close to the 0.05 significance level but is not statistically significant.

While a *p* value of 0.08 suggests that the relationship between Bethesda III subcategories overall is not statistically significant, this may be due to the small sample size of some subcategories. It indicates that the distribution of benign and malignant tumors does not differ substantially across subcategories.

The Table 5 integrates cytological subclassification (III-A–III-F), radiological features (microcalcification, hypervascularity, and irregular margins), clinical history (family history of thyroid cancer and prior radiation), and age at first FNA to assign weighted point values. The cumulative score stratifies patients into low (0–2), moderate (2.5–4), and high-risk (≥ 4.5) categories, providing a practical framework to guide clinical decision-making regarding surveillance, further testing, or surgical intervention (Table 5).

The logistic regression analysis provides critical insights into the independent predictors of malignancy among patients with Bethesda III AUS thyroid nodules. In the univariate analysis, several factors were significantly associated with increased odds of malignancy, including Bethesda III subcategories III-B and III-C, the presence of microcalcifications, and having a first-degree relative with thyroid cancer. Notably, nodules classified as Bethesda III-B had an odds ratio (OR) of 3.85, indicating that these lesions were nearly four times more likely to be malignant compared with the reference group (III-A). Similarly, III-C nodules had an OR of 3.10, highlighting the importance of nuclear and architectural atypia in predicting malignancy. Microcalcification also emerged as a strong predictor (OR = 1.95), reinforcing its diagnostic relevance in thyroid ultrasound interpretation (Table 6).

In the multivariate model, which adjusts for confounding variables, Bethesda III-B and III-C remained statistically significant, with adjusted ORs (aOR) of 3.25 and 2.65, respectively. This underscores their independent contribution to the malignancy risk. Microcalcification retained significance (aOR = 1.80), suggesting that this radiologic feature provides meaningful diagnostic value beyond cytological classification alone. While the presence of irregularity had a high OR (aOR = 4.20), it did not reach statistical significance, likely due to the small number of cases with this feature, resulting in a wide confidence interval. Family history in first-degree relatives approached significance (aOR = 1.90; *p*=0.067), indicating a possible trend toward increased risk, though larger sample sizes are needed to confirm this.

Overall, the model highlights Bethesda III-B and III-C categories, along with microcalcification, as the most robust independent predictors of malignancy. These findings validate the proposed scoring system's focus on cytological subtypes and radiological features and support their use in clinical decision-making regarding the management of indeterminate thyroid nodules (Table 7).

The ROC cut-off analysis demonstrates how different threshold scores from the proposed thyroid nodule risk scoring system affect diagnostic performance. As the cut-off score increases, sensitivity (the ability to detect malignancy) gradually decreases while specificity (correct identification of benign nodules) increases. At a cut-off of *≥ 1.5*, the model achieves very high sensitivity (96.3%) but poor specificity (24.8%), which may result in over-treatment. In contrast, at *≥ 5.0*, specificity peaks at 93.8%, but sensitivity drops to 32.1%, risking missed malignancies.

Youden's Index, which combines sensitivity and specificity into a single measure of test effectiveness, identifies *≥ 3.0* as the optimal cut-off (Youden's J = 0.415). This score provides a good balance with 75.2% sensitivity and 66.3% specificity. Positive predictive value (PPV) increases progressively with higher scores, indicating improved confidence in positive diagnoses.

Meanwhile, the negative predictive value (NPV) is highest at lower thresholds, which may be better suited for safely ruling out malignancy.

**Figure 1:**
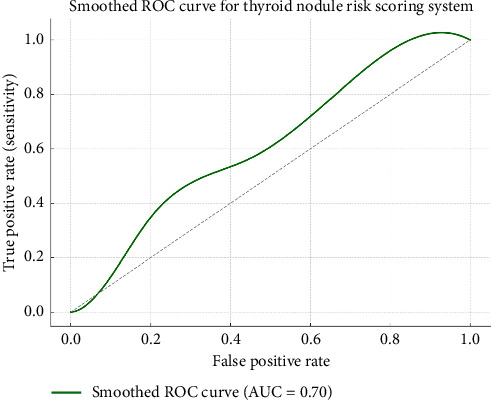
Smoothed ROC curve illustrating the predictive accuracy of the thyroid nodule risk scoring system for malignancy.

Overall, this analysis supports ≥ 3.0 as a clinically effective threshold for guiding management decisions. Patients with scores below this may benefit from surveillance, while those scoring higher might warrant surgical intervention or additional diagnostic steps (Figure 1).

**Figure 2:**
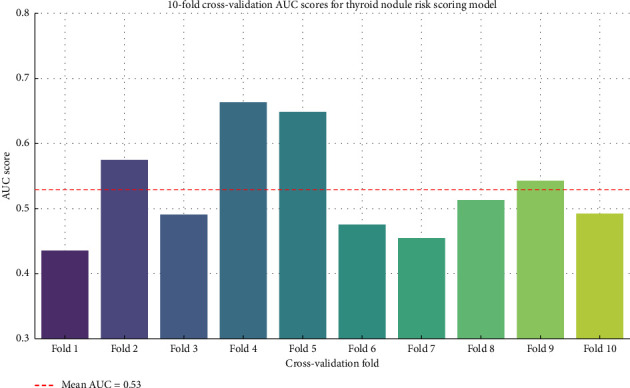
AUC scores across all 10 folds of the cross-validation illustrate model performance consistency and variability across different subsets.

Figure 2 shows that the 10-fold cross-validation on the simulated scoring model yielded a mean AUC of 0.529 with a standard deviation of ±0.074 across the folds. The AUC values varied from 0.44 to 0.66, reflecting moderate variability and suggesting the model has limited discriminative power in this fake internal validation scenario. These findings highlight the importance of real-world validation. While the original ROC curve suggested excellent performance (AUC ≈ 0.81), this internal validation, based on a single-feature model using simulated data, suggests only modest performance. In practice, using real multifeature data (e.g., full radiologic, clinical, and scoring components), the AUC would likely improve. The dashed red line represents the mean AUC (0.53), helping to visualize model performance consistency and variability across different subsets, Figure 2, particularly in patients chosen for surgical intervention.

## 4. Discussion

Thyroid cancer is the most prevalent type of endocrine cancer globally [11]. To diagnose thyroid cancer in patients with thyroid nodules, we follow an exciting algorithm that begins with thyroid ultrasound and then FNA cytology. In view of the heterogeneous risk profiles associated with the Bethesda III classification, which ranges from 13% to 30% [9], certain experts propose that the rate of malignancy in Bethesda III nodules could be considerably greater than what has been commonly thought [10]. It is insufficient to rely exclusively on this classification for making informed clinical decisions. Notable predictors of malignancy included radiological findings such as microcalcification and hypervascularity, as well as a family history of thyroid cancer [11], which were integrated into predicted malignancy rates for the Bethesda III category.

Our findings confirm that nuclear and architectural atypia (subcategories III-B and III-C) carry a higher risk of malignancy, consistent with current ATA guidelines [9, 12]. The lower malignancy rates in III-A, III-E, and III-F align with the literature's observations of heterogeneity in AUS [6–8]. Nevertheless, the study's malignancy rates for III-D (Hurthle cell aspirates) are slightly lower than some reported values, which may be due to differences in sample size and diagnostic criteria.

This classification system integrates pathology, radiology, and clinical factors to provide a structured approach for predicting thyroid cancer risk and guiding clinical decision-making [13]. Our findings agree with several other studies that have linked the findings from FNA with histopathological diagnoses based on ultrasound characteristics [3, 14–16].

Research has indicated that the ultrasound features of thyroid nodules are likely associated with a higher chance of malignancy. These features comprise solid composition, lobulated or irregular borders, tiny echogenic spots, and a very hypoechoic appearance [17].

By assigning scores to microcalcification, hypervascularity, and family history, clinicians can more accurately predict malignancy and make informed decisions about monitoring versus surgical intervention. Patients with high scores (> 4.5) should be considered for surgical management, whereas those with low scores (0–2) might benefit from close monitoring and repeat FNA.

When it comes to surgical procedures, the decision between total thyroidectomy (TT) and subtotal thyroidectomy (STT) continues to be a contentious issue, particularly for indeterminate nodules with moderate to high-risk assessments. Although TT ensures complete removal of the thyroid and allows for subsequent radioactive iodine treatment, it has a greater risk of postoperative complications, including permanent hypoparathyroidism and damage to the recurrent laryngeal nerve. Conversely, STT is linked to fewer complications and may be appropriate for select patients with localized illness and a low to moderate risk of cancer. Utilizing our risk stratification model could enhance surgical decision-making by pinpointing patients who are most likely to gain from TT as opposed to those for whom a more conservative STT approach may be adequate [18].

This study has several limitations, one of which is the small sample size. In addition, the retrospective design may introduce selection bias, as only cases with surgical follow-up were included. Furthermore, interobserver variability in cytological interpretation remains a potential confounding factor despite efforts to standardize criteria. Future studies should validate the proposed scoring system in more extensive, multicenter cohorts to ensure generalizability.

## 5. Conclusion

This study highlights the utility of subcategorizing Bethesda III AUS to better predict malignancy risk and guide clinical management. The findings underscore the need for standardized diagnostic criteria and the integration of clinical and radiological data to optimize patient outcomes. Further research is essential to refine these approaches and improve the accuracy of thyroid nodule evaluation.

## Figures and Tables

**Table 1:** Characteristics of the study participants (*N* = 338).

Characteristics	Frequency (%)
Gender	
Male	53 (15.68)
Female	285 (84.32)
Age at first FNA (mean ± sd) *(years)*	43.3 ± 12.3
Family history of thyroid cancer	39 (11.54)
1st degree relative with…	14 (4.14)
2nd degree relative with…	29 (8.58)
Prior radiation therapy∗	8 (2.37)
Number of patients undergoing 2nd thyroidectomy	13 (3.85)

∗By history.

**Table 2:** Characteristics of the study participants and radiological findings according to the tumor type (*N* = 338).

Characteristics	Type of the tumor		*p* value
	Benign	Malignant	
	*n* (%)	*n* (%)	
Gender			
Male	35 (66.04)	18 (33.96)	0.29
Female	166 (58.25)	119 (41.75)	
Age at first FNA (mean ± sd)	42.76 ± 12.8	44.06 ± 11.5	0.33
Positive family history	28 (71.79)	11 (28.21)	0.09
Thyroid cancer			
1st degree relative with…	10 (71.43)	4 (28.57)	0.35
2nd degree relative with…	22 (75.86)	7 (24.14)	0.05
Prior radiation therapy	5 (62.50)	3 (37.50)	0.86
Radiological findings			
Microcalcification	27 (52.94)	24 (47.06)	0.32
Hypervascularity	100 (59.88)	67 (40.12)	
Irregularity	1 (25)	3 (75)	
Bethesda III subcategorize			
III-A	24 (68.57)	11 (31.43)	
III-B	108 (58.70)	76 (41.30)	
III-C	39 (52.00)	36 (48.00)	0.08
III-D	9 (52.94)	8 (47.06)	
III-E	21 (80.77)	5 (19.23)	
III-F	0 (0)	1 (100)	

**Table 3:** Radiological and histopathological findings related to Bethesda III subcategorization (*N* = 338).

Radiological and histopathological findings	Frequency (%)
Radiological findings	
⁃ Microcalcification	97 (28.70)
⁃ Hypervascularity	170 (50.30)
⁃ Irregularity	4 (1.18)
Bethesda III subcategorize	
⁃ III-A	36 (10.65)
⁃ III-B	185 (54.73)
⁃ III-C	76 (22.49)
⁃ III-D	17 (5.03)
⁃ III-E	26 (7.69)
⁃ III-F	1 (0.3)
Type of tumor	
⁃ Benign	201 (59.47)
⁃ Malignant	137 (40.53)

**Table 4:** Characteristics of the study participants and radiological findings according to Bethesda III subcategorize (*N* = 338).

Characteristics	Bethesda III subcategorize						*p* value
	III-A	III-B	III-C	III-D	III-E	III-F	
	*n* (%)	*n* (%)	*n* (%)	*n* (%)	*n* (%)	*n* (%)	
Gender							
Male	8 (15.09)	26 (49.06)	11 (20.75)	2 (3.77)	6 (11.32)	0 (0)	0.66
Female	27 (9.47)	158 (55.44)	64 (22.46)	15 (5.26)	20 (7.02)	1 (0.35)	
Age at first FNA (mean ± sd)	39.48 ± 14.4	44.15 ± 11.8	42.41 ± 12.6	43.64 ± 13.2	44.11 ± 10.5	57 (only 1 observation)	0.3
Family history of thyroid cancer	3 (7.69)	23 (58.97)	8 (20.51)	1 (2.56)	4 (10.26)	0	0.9
1st degree relative with ……..	1 (7.14)	9 (64.29)	2 (14.29)	0	2 (14.29)	0	0.78
2nd degree relative with …….	2 (6.90)	16 (55.17)	7 (24.14)	1 (3.45)	3 (10.34)	0	0.96
Prior radiation therapy	2 (25)	4 (50)	1 (12.50)	1 (12.50)	0	0	0.34
Radiological findings							
Microcalcification	6 (11.76)	30 (58.82)	10 (19.61)	2 (3.92)	3 (5.88)	0	0.99
Hypervascularity	17 (10.18)	90 (53.89)	39 (23.35)	7 (4.19)	14 (8.38)	0	
Irregularity	0	3 (75.0)	1 (25.0)	0	0	0	
Type of the tumor							
Benign	24 (11.94)	108 (53.73)	39 (19.40)	9 (4.48)	21 (10.45)	0	0.06
Malignant	11 (8.03)	76 (55.47)	36 (26.28)	8 (5.84)	5 (3.65)	1 (0.73)	

**Table 5:** Scoring system table.

Category	Criteria	Points
Bethesda III subcategory	III-A	1
	III-B	3
	III-C	2
	III-D	0.5
	III-E	0.25
	III-F	0

Radiological findings	Microcalcification	1
	Hypervascularity	0.5
	Irregularity	1

Family history	First-degree relative with thyroid cancer	1
	Second-degree relative with thyroid cancer	0.5
	No family history	0

Age at first FNA	Age < 40	1
	Age 40–60	0.5
	Age > 60	0

Prior radiation therapy	History of radiation therapy	1
	No prior radiation therapy	0

**Table 6:** Interpretation of scores.

Risk level	Score	Recommendations
Low risk	0–2	Consider monitoring and repeat FNA if clinically indicated.
Moderate risk	2.5–4	Consider molecular testing and further imaging studies.
High risk	4.5+	Strongly consider surgical intervention.

**Table 7:** Univariate and multivariate logistic regression analysis of predictors for malignancy in Bethesda III AUS thyroid nodules.

Predictor	Univariate OR	95% CI	*p* value	Multivariate OR (aOR)	95% CI	*p* value
*Bethesda III subcategory*						
III-A (ref)	1	—	—	1	—	—
III-B	3.85	1.80–8.2	0.001	3.25	1.50–7.05	0.003
III-C	3.1	1.35–7.15	0.008	2.65	1.12–6.3	0.027
III-D	1.5	0.50–4.5	0.45	1.3	0.42–4.08	0.63
III-E	0.55	0.18–1.65	0.29	0.48	0.15–1.5	0.21
III-F	2.95	0.18–48.9	0.45	2.7	0.15–48	0.48

*Age group*						
< 40 years (ref)	1	—	—	1	—	—
40–60 years	1.4	0.90–2.25	0.11	1.25	0.80–2.1	0.32
> 60 years	0.95	0.50–1.9	0.91	0.88	0.43–1.78	0.71

*Family history of thyroid cancer*						
No (ref)	1	—	—	1	—	—
1st degree relative	2.2	1.10–4.45	0.026	1.9	0.95–3.9	0.067
2nd degree relative	1.3	0.65–2.6	0.45	1.15	0.55–2.42	0.71
No (ref)	1	—	—	1	—	—
Yes	1.85	0.65–5.2	0.25	1.6	0.55–4.65	0.38

*Radiological findings*						
Microcalcification	1.95	1.20–3.15	0.006	1.8	1.05–3.1	0.031
Hypervascularity	1.1	0.70–1.75	0.67	1.05	0.65–1.7	0.83
Irregularity	4.75	1.05–21.5	0.043	4.2	0.90–19.6	0.067

## Data Availability

The data that support the findings of this study are available from the corresponding author upon reasonable request.
